# Transcriptome Analysis of *Acinetobacter baumannii* in Rapid Response to Subinhibitory Concentration of Minocycline

**DOI:** 10.3390/ijerph192316095

**Published:** 2022-12-01

**Authors:** Lili Gao, Xiaochun Ma

**Affiliations:** 1 College of Grassland Science, Qingdao Agricultural University, Qingdao 266109, China; 2 Experimental Animal Center, Zunyi Medical University, Zunyi 563003, China

**Keywords:** *A. baumannii*, minocycline, transcriptome analysis, antibiotic resistance, adaptive response

## Abstract

The increasing emergence of multidrug-resistant *Acinetobacter baumannii* brings great threats to public health. Minocycline is a kind of semisynthetic derivative of the antibacterial drug tetracycline and is often used to treat infections caused by multidrug-resistant *A. baumannii* with other antibiotics. However, minocycline-resistant *A. baumannii* appears constantly. To rapidly explore the response of *A. baumannii* to minocycline stress, RNA-seq was carried out to compare the difference in the transcriptome of *A. baumannii* ATCC19606 in the presence or absence of minocycline. The results showed that 25 genes were differentially expressed, including 10 downregulated genes and 15 upregulated genes, and 24 sRNA were upregulated and 24 were downregulated based on the filter criteria (Log2FC > 1 or <−1 and FDR < 0.05). RtcB family protein and ABC transporter ATP-binding protein were upregulated by 2.6- and 11.3-fold, and molecular chaperone GroES, chaperonin GroL, class C beta-lactamase ADC-158, amino acid ABC transporter permease, and APC family permease were downregulated by at least two-fold in the presence of half-MIC minocycline. The differentially expressed genes are mainly involved in the stress response, the GroES/GroEL chaperonin system, and transport metabolic pathways. sRNA 1248 was significantly upregulated, and sRNA 1767, 5182, and 6984 were downregulated in a rapid response to minocycline. These results provide insights into the adaptive mechanism of *A. baumannii* to minocycline.

## 1. Introduction

*Acinetobacter baumannii* (*A. baumannii*) is an aerobic, nonfermentative, Gram-negative coccobacilli. It is ubiquitous in natural environments, such as water and soil, and hospital environments [1]. The bacteria inhabit the skin, respiratory tract, and urinary tract of the body and are widely isolated from specimens of animal origins, such as swine, dogs, cats, horses, and so on [2]. As an important opportunistic pathogen in hospitals, a variety of infections, including ventilator-associated pneumonia, urinary tract infections, skin and soft tissue infections, wound infections, and bloodstream infections, are caused by *A. baumannii* [3]. The emergence of multidrug-resistant (MDR) *A. baumannii* brings a great threat to people. *A. baumannii* has become one of the top 10 clinical bacteria in children’s hospitals in China [4]. A survey concerning inpatients with Gram-negative bloodstream infections between 2009 and 2013 reported that the proportion of difficult-to-treat resistance of *A. baumannii* was 18.3% [5].

Adaptive and protective responses are induced when bacteria are in a stressful environment, which impacts the susceptibility of bacteria to antimicrobials. Antimicrobials themselves are important stressors, leading to the promotion of resistance [6]. Minocycline is a kind of semisynthetic derivative of the antibacterial drug tetracycline, binding to the 30S subunit of ribosomes to prevent the extension of peptide chains and inhibit the synthesis of proteins of pathogens [7]. It was used orally and intravenously to treat infections caused by *A. baumannii* [8]. Due to the disadvantages of colistin monotherapy, a combination therapy with minocycline is gradually used to treat infections caused by MDR *A. baumannii* [9]. Synergistic effects of the combination therapy of colistin with minocycline for pneumonia caused by MDR *A. baumannii* were observed [10]. The Tigecycline Evaluation and Surveillance Trial (TEST) study reported an 84.5% susceptibility rate to minocycline between 2004 and 2013 [11]. The susceptibility of the *A. baumannii*–*A. calcoaceticus* species complex to minocycline collected from U.S. hospitals from 2014 to 2018 was 85.7% [12]. The resistance of minocycline is related to the tetracycline efflux by membrane-related proteins and resistance-nodulation-division (RND)-type efflux pumps [11]. TetB was demonstrated to reduce the susceptibility to minocycline in *A. baumannii* [13].

The strain ATCC19606 is one of the best-characterized strains of *A. baumannii*, which is widely used to study antimicrobial resistance and other stress [14,15,16]. It is resistant to sulfonamide but susceptible to a variety of other antibiotics, including β-lactams, aminoglycosides, quinolones, tetracyclines, and colistin [17,18]. In the present study, to learn the adaptive response of *A. baumannii* to antibiotics, the transcriptome of ATCC19606 cells exposed to a subinhibitory concentration of minocycline was analyzed. Our results revealed differentially expressed genes involved in the stress response, ABC transporters, the GroES/GroEL chaperonin system, beta-lactam resistance, the degradation of aminobenzoate, valine, leucine, and isoleucine, the biosynthesis of secondary metabolites, and metabolic pathways as well as sRNA involved in acute regulation in response to minocycline exposure for a short time. These findings provide a basis for minocycline resistance and target genes for potential therapies.

## 2. Materials and Methods

### 2.1. Bacterial Strains, Growth Conditions, and Susceptibility Testing

The MIC of *A. baumannii* strain ATCC19606 to minocycline was determined by the broth microdilution method according to the Clinical and Laboratory Standard Institute (CLSI, 2020) guidelines [19]. Briefly, bacteria grown in Müller–Hinton (MH) broth was adjusted to level 0.5 of the McFarland scale. Minocycline was twofold-diluted successively on 96-well plates. Background blank and positive controls were included in each experiment. Then, 100 μL of a bacterial suspension was added and incubated at 37 °C for 24 h. The lowest concentration with no visible bacteria growth was considered the MIC. 

*A. baumannii* strain ATCC19606 was cultured in Luria–Bertani (LB) medium at 37 °C. The overnight culture was inoculated into LB broth in the proportion of 1:100. Minocycline was added into the medium until the OD600 of the culture reached 0.6 at 37 °C. Sterile water was used in the control. 

### 2.2. Total RNA Extraction and rRNA Removal

The bacterial cells were harvested by centrifugation after incubation with half-MIC minocycline for 10 min. The bacteria were washed with precooled PBS and digested with lysozyme at 37 °C for 15 min. The pellets were collected by centrifugation at 5000 rpm for 20 min and subjected to grinding in liquid nitrogen at a low temperature. After sufficient grinding, the MasterTM RNA Purification Kit was applied to extract the total bacterial RNA, according to the kit instructions. Subsequently, rRNA was removed from the total RNA using the Ribo-zeroTM rRNA Depletion Kit (Epicentre: Madison, WI, USA), and the RNA quality was detected using an Agilent2200 biological nucleic acid analyzer.

### 2.3. High-Throughput Sequencing cDNA Library Construction and RNA-Seq Sequencing

A mixture of mRNA and ncRNA was randomly cut into short fragments from 100 to 300 nt. A sequencing adaptor was added to the fragments according to the requirements of library construction kits for the Illumina sequencing platform. Then, the samples were subjected to high-throughput sequencing on an Illumina HiSeq 4000 sequencer. 

### 2.4. Transcriptome Analysis

Fast-QC software was used to evaluate the overall quality of the sequencing data, including the distribution of base mass values and position, GC content, PCR duplication content, frequency of kmer, etc. HISAT2 software was used to compare the RNA-seq data, and the filtered clean reads were mapped. The differential expression of genes was screened based on log2FC > 1 or <−1 and FDR < 0.05, according to the DESeq2 algorithm. COG and pathway analyses of differentially expressed genes were performed based on the COG and gene annotation database.

### 2.5. Candidate sRNA Identification and RT-qPCR Validation

The sRNA of the samples was predicted based on the original sequencing data of this project, according to the default parameters of the sRNA kit algorithm. The location of sRNA was annotated on the reference genome, and the expression was quantified. Differentially expressed sRNA was screened based on log2FC > 1 or <−1 and FDR < 0.05, according to the DESeq2 algorithm.

The top 15 differentially expressed sRNAs were selected for validation. The primers of candidate sRNAs were designed. The bacterial pellets were resuspended in TRIzol reagent (Invitrogen, Waltham, MA, USA). The total RNA of samples was extracted as previously reported [13]. The RNA pellet was resuspended with DEPC water. cDNA was reverse-transcribed with a PrimeScript™ RT reagent Kit with gDNA Eraser (Takara: Dalian, China), which was then used for quantitative real-time PCR (qPCR) with TB Green™ Premix Ex Taq™ II (Takara: Dalian, China). The qPCR was performed on a CFX96 Real-Time PCR Detection System (Bio-Rad Laboratories: Hercules, CA, USA).

### 2.6. Statistical Analysis

Data were analyzed by Student’s *t*-test. The statistical analysis was performed using GraphPad Prism software (verson 8.0, San Diego, CA, USA). A *p* value below 0.05 was considered significant. The qPCR data were analyzed using CFX maestro software (1.0). A *p* value below 0.01 was considered significant in qPCR.

## 3. Results

### 3.1. Transcriptome Analysis of A. baumannii Strain ATCC19606

The minimum inhibitory concentration (MIC) of ATCC19606 to minocycline was 2 μg/mL. To uncover the role that sRNA and mRNA of *A. baumannii* play in the exposure to antibiotics, *A. baumannii* strain ATCC19606 was subjected to RNA-seq after incubation in the presence of a subinhibitory concentration of minocycline and a control. After quality control and de novo sequencing, HISAT2 software was used for mapping and data comparisons.

The RNA sequencing results showed that a total of 72~81 million reads were acquired when *A. baumannii* ATCC19606 was stimulated by half-MIC minocycline, and 64~73 million reads were mapped to the *A. baumannii* genome. Among the 63~77 million reads in the control, 56~69 million reads were mapped. The reads were mainly distributed in NZ_CZWC01000006.1, followed by NZ_CZWC010000011.1 and NZ_CZWC01000002.1. A gene structure analysis showed that the detected reads were mainly located on exons and CDS regions, followed by 5′-UTRs and intergenic regions. Compared with the control, more reads were detected on 5′-UTRs and less reads were detected on CDS regions.

### 3.2. Differentially Expressed mRNA and sRNA

**Figure 1:**
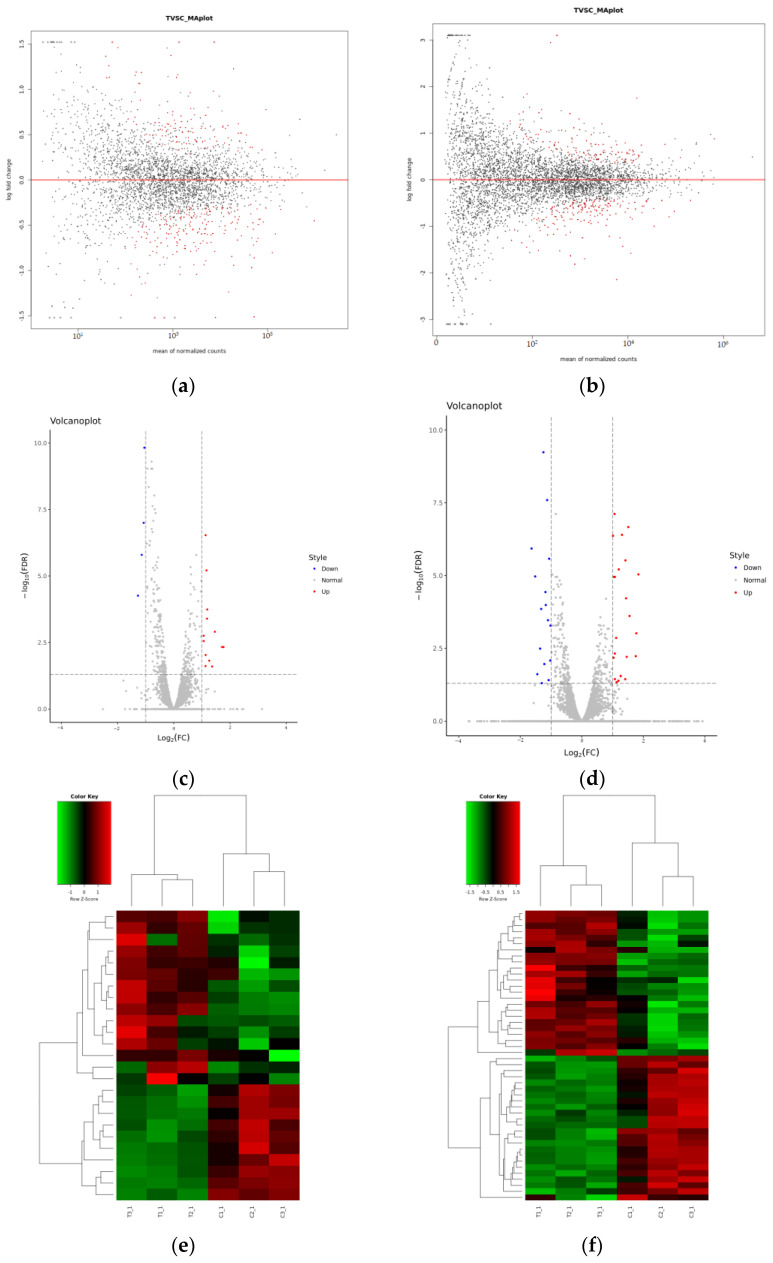
(**a**) MAplot of genes detected in response to minocycline. (**b**) MAplot of detected sRNA. Differentially expressed genes and sRNA are shown in red. (**c**) Volcano plot of differentially expressed genes (DGEs) of *A. baumannii* in the presence or absence of minocycline. (**d**) Volcano plot of differentially expressed sRNA. Upregulated or downregulated genes and sRNA with a log2-fold change ≥ 1 are shown in red and blue. (**e**) Expression heat map of DGEs. (**f**) Expression heat map of differentially expressed sRNA.

In our research, genes of differential expression were also included. Desequ2 was used to screen the differentially expressed counts based on the filter criteria (Log2FC > 1 or <−1 and FDR < 0.05). When *A. baumannii* ATCC19606 were cultured in a subinhibitory concentration of minocycline for 10 min, a total of 3220 genes were detected by RNA sequencing (Figure 1a,e), while 7317 sRNAs were obtained when in solution (Figure 1c). According to the filter criteria, a total of 25 genes were differentially expressed, including 10 downregulated genes and 15 upregulated genes (Table 1). 

Of the 48 differentially expressed sRNAs, 24 sRNAs were upregulated and 24 sRNAs were downregulated based on the filter criteria (Log2FC > 1 or <−1 and FDR < 0.05) after incubation in a subinhibitory concentration of minocycline for 10 min (Figure 1b,d,f).

### 3.3. Functional Annotation of Differentially Expressed Genes

**Figure 2:**
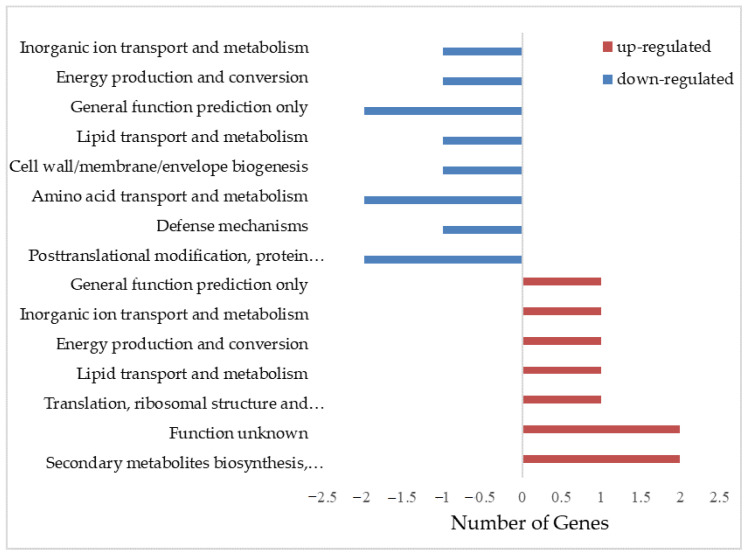
Cluster of orthologous genes (COG) classification of differentially expressed genes.

A COG (clusters of orthologous groups of proteins) analysis was carried out to evaluate the functional categorization. The results showed that the differentially expressed genes were classified into 11 COG categories. The 15 upregulated genes were involved in secondary metabolite biosynthesis, transport and catabolism, translation, ribosomal structure and biogenesis, lipid transport and metabolism, energy production and conversion, inorganic ion transport and metabolism, function unknown, and general function prediction. The 10 downregulated genes were involved in post-translational modification, protein turnover, chaperones, defense mechanisms, amino acid transport and metabolism, cell wall/membrane/envelope biogenesis, lipid transport and metabolism, general function prediction, energy production and conversion, and inorganic ion transport and metabolism (Figure 2). 

A KEGG pathway analysis revealed that the differentially expressed genes were linked to 17 pathways. The upregulated genes were involved in styrene degradation; the biosynthesis of siderophore group nonribosomal peptides; aminobenzoate degradation; arginine and proline metabolism; nicotinate and nicotinamide metabolism; tryptophan metabolism; phenylalanine metabolism; the biosynthesis of antibiotics; energy metabolism; and the biosynthesis of secondary metabolites, while the downregulated genes were involved in inositol phosphate metabolism; the two-component system; RNA degradation; beta-alanine metabolism; beta-lactam resistance; valine, leucine, and isoleucine degradation; propanoate metabolism; sulfur metabolism; ABC transporters; and carbon metabolism. Furthermore, *rtcB*, which is involved in stress response, was upregulated. Two downregulated genes (*groS* and *groL*) were involved in the GroES/GroEL chaperonin system.

### 3.4. Genes Upregulated by Minocycline Treatment

The upregulated genes encoded a putative methionine/alanine importer small subunit (A6739_RS00130), RtcB family protein (A6739_RS02970), 2,3-dihydroxybenzoate-AMP ligase (*entE*), hypothetical protein (A6739_RS05460, A6739_RS06410, A6739_RS07175, A6739_RS15535, A6739_RS11675 and A6739_RS10785), TonB-dependent siderophore receptor (A6739_RS06520), ABC transporter ATP-binding protein (A6739_RS06535), amidase (A6739_RS07555), acyl-CoA dehydrogenase (A6739_RS07560), aromatic-ring-hydroxylating dioxygenase subunit beta (A6739_RS07580), and NAD(P) transhydrogenase subunit alpha (A6739_RS14015).

### 3.5. Genes Downregulated by Minocycline Treatment

Genes encoding molecular chaperone GroES (A6739_RS03845), chaperonin GroL (A6739_RS03850), class C beta-lactamase ADC-158 (A6739_RS05090), methylmalonate-semialdehyde dehydrogenase (CoA acylating) (A6739_RS05735), FMN-dependent NADH-azoreductase (A6739_RS08375), TIGR01244 family phosphatase (A6739_RS08405), MBL fold metallo-hydrolase (A6739_RS08410), amino acid ABC transporter permease (A6739_RS09455), APC family permease (A6739_RS11485) and copper resistance protein NlpE (A6739_RS12210) were downregulated.

### 3.6. Validation of sRNA

**Figure 3:**
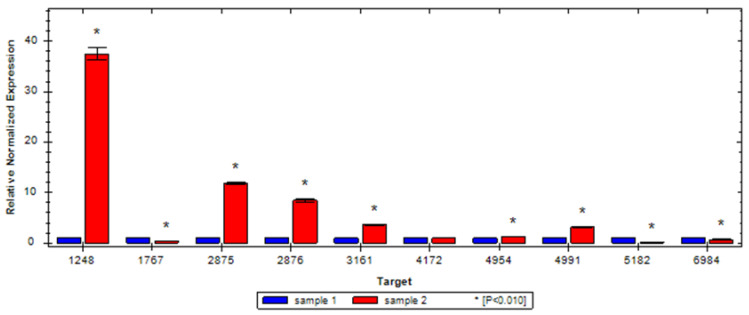
Validation of differentially expressed sRNAs. sample 1, control; sample 2, *A. baumannii* after exposure to subinhibitory minocycline.

Fifteen sRNAs, 1248, 5182, 4806, 6534, 6535, 6543, 6544, 1767, 2875, 2876, 3161, 4172, 4954, 4991, and 6984, were selected from the differentially expressed sRNA for validation by qRT-PCR. Of the tested sRNAs, 1248, 2875, 2876, 3161, and 4991 were upregulated, which was in agreement with the RNA-seq results (Figure 3). A significant 27-fold upregulation was observed in sRNA 1248. sRNAs 1767, 5182, and 6984 were significantly downregulated, in accordance with the results of RNA-seq. There was no significant change in sRNA 4172 or 4954 between the control and experimental groups. 

## 4. Discussion

Due to the use of antibiotics in human and veterinary medicine, subinhibitory concentrations of antibiotics have been detected in hospital effluent, municipal sewage, the effluent of sewage treatment plants, surface water, and ground water [6,20]. Exposure to subinhibitory concentrations of antibiotics is thought to increase the evolution speed of bacteria, leading to resistant strains [21]. Minocycline is one member of the tetracycline family, and it acts by inhibiting protein synthesis in bacteria. It not only plays roles in the treatment of some skin and sexually transmitted diseases but also has biological actions such as anti-inflammatory and antiapoptotic activities [9,22]. Minocycline is approved for the intravenous treatment of infections caused by multidrug-resistant *A. baumannii*. In this study, to explore the rapid response of *A. baumannii* in the presence of minocycline, RNA-seq was performed when *A. baumannii* was exposed to half-MIC minocycline.

RtcB is a conserved protein that exists widely in bacteria, archaea, protozoa, and metazoans [23]. It can splice the 5′-OH ends of RNA to either 2′, 3′-cyclic phosphate or 3′-phosphate ends to repair RNA damage to recover from stress-induced RNA damage in response to cellular stress [24,25]. In *Escherichia coli* (*E. coli*), the RNA ligase RtcB religates the truncated 16S rRNA to ribosomes to restore their ability for the recovery of *E. coli* upon stress release [26]. In response to a low concentration of minocycline, the gene encoding RtcB family protein was upregulated rapidly in this study, which suggested that RtcB responded rapidly in the presence of minocycline to repair the RNA damage induced by antibiotic exposure. Class C beta-lactamase ADC-158, as a member of the beta-lactamases, was downregulated in response to minocycline. The downregulation of b-lactamases such as OXA-23 and AmpC was also observed in the presence of tigecycline [26]. The results may give the explanation that a combination therapy with minocycline is more effective to treat MDR *A. baumannii*. ABC transporters are ubiquitous in bacteria, functioning in importing nutrients and exporting antimicrobial agents [27]. Several studies reported that the ABC transporter system was inhibited in the presence of antibiotics such as sulbactam and tigecycline [27,28]. In this study, APC family permease was also downregulated. The ABC transporter consumed ATP to transport toxins or antimicrobial agents, which may be one of the resistance mechanisms to antibiotics. The GroES/GroEL chaperonin system is highly conserved across species and is essential in various environments [29]. GroES assists GroEL to encapsulate and fold unfolded polypeptides in a privileged hydrophilic chamber sequestered from the cellular milieu [30]. GroEL/ES inhibitors were considered as potential antibiotics to treat bacterial infections [31,32]. It was reported that chaperonin GroL/GroES was upregulated in response to stress (heat shock, antibiotics, and oxidative stress) [33]. The overexpression of the chaperonin GroEL/GroES system promotes short-term tolerance to aminoglycosides in *E. coli* and Mycobacterium abscessus [34,35]. However, both chaperonin GroEL and GroES were downregulated in our study. Chaperone Hsp60 (GroLS) was also downregulated in the presence of tigecycline [28]. The differential expression of chaperonin GroL/GroES may be related with the type of antibiotics and could be responsible for protection from minocycline.

Bacterial noncoding RNAs (sRNAs) are a group of RNAs that are transcribed from intergenic regions, untranslated regions, or even some open reading frames with lengths from 40 nt to 500 nt [36]. sRNA mainly acts as a regulator at the post-transcriptional level for bacteria to adapt to environmental changes. At low temperatures, sRNA DsrA was transcribed to bind with rpoS mRNA to activate the translation of the stationary-phase sigma factor RpoS in *E. coli* [37]. sRNA AbsR25 of *A. baumannii* responded to ethidium bromide [38]. The overexpression of sRNA 13573 in *A. baumannii* contributed to the formation of biofilm and helped the bacteria to adhere to human alveolar epithelial cells [39]. 

An increasing number of sRNAs were gradually discovered and identified from Gram-negative and -positive bacteria working as post-transcriptional regulators in response to stress [40]. sRNA plays a major role of the regulation of adaptation to antibiotic stress [41]. The expression level of sRNA changed significantly, and it conferred advantages to bacterial survival when bacteria were challenged with different kinds of antibiotics. *S. typhimurium* produced more sRNA sYJ20 in the presence of ciprofloxacin, tigecycline, or tetracycline [36]. SprX influenced glycopeptide resistance by negatively regulating SpoVG expression in *Staphylococcus aureus* [42]. Once it encounters antibiotics, sRNA is mobilized rapidly to introduce response kinetics into regulatory circuits for adaptation [43]. Class I sRNAs are involved in tight transcriptional regulation in emergency stress and disappear rapidly when no transcription signals are received [44,45]. A profile of sRNA from methicillin-resistant *Staphylococcus aureus* (MRSA) after exposure to several antimicrobial agents for 10 min was obtained to regulate antibiotic stress [46]. In this study, 48 sRNAs were discovered from *A. baumannii* after exposure to minocycline, which indicated that sRNA was rapidly mobilized in the presence of minocycline to regulate the expression of mRNA. sRNA 1248 was significantly upregulated and may be the important regulator in response to minocycline stress. However, the functions of sRNA and the regulation mechanism were not explored and need to be further studied.

## 5. Conclusions

The transcriptional profile of *A. baumannii* in the presence or absence of minocycline was analyzed. The results showed that 25 genes and 48 sRNAs were differentially expressed. RtcB family protein and ABC transporter ATP-binding protein were upregulated, and chaperonin GroL, class C beta-lactamase ADC-158, and ABC transporter ATP-binding protein were significantly downregulated in the presence of half-MIC minocycline. The results revealed differentially expressed genes involved in the stress response, ABC transporters, the GroES/GroEL chaperonin system, beta-lactam resistance, the degradation of aminobenzoate, valine, leucine, and isoleucine, the biosynthesis of secondary metabolites, and metabolic pathways in response to minocycline exposure for a short time. sRNA 1248 was significantly upregulated, and sRNAs 1767, 5182, and 6984 were downregulated in a rapid response to minocycline. These sRNAs may be the key regulators. The functions of these sRNAs need to be further studied as a resistance mechanism to minocycline.

## Figures and Tables

**Table 1:** Differentially expressed genes in the presence of minocycline.

AccID	Gene Name	log2FC	Style	Description	Molecular Function
A6739_RS00130	-	1.261057061	up	putative methionine/alanine importer small subunit	-
A6739_RS02970	*rtcB*	1.375621078	up	RtcB family protein	GTP binding, metal ion binding, RNA ligase
A6739_RS03845	*groS*	−1.587812857	down	molecular chaperone GroES	ATP binding, ATP-dependent protein folding chaperone, isomerase, unfolded protein binding
A6739_RS03850	*groL*	−1.236322333	down	chaperonin GroL	ATP binding, ATP-dependent protein folding chaperone, isomerase, unfolded protein binding
entE	*entE*	1.133073343	up	“2,3-dihydroxybenzoate-AMP ligase”	(2,3-dihydroxybenzoyl) adenylate synthase, ATP binding, ligase
A6739_RS05090	*blaADC*	−1.511731996	down	class C beta-lactamase ADC-158	beta-lactamase
A6739_RS05460	-	1.190591988	up	hypothetical protein	-
A6739_RS05735	*mmsA*	−1.046110868	down	methylmalonate-semialdehyde dehydrogenase (CoA acylating)	malonate-semialdehyde dehydrogenase (acetylating), methylmalonate-semialdehyde dehydrogenase (acylating)
A6739_RS06410	-	1.065451376	up	hypothetical protein	-
A6739_RS06520	-	1.183871644	up	TonB-dependent siderophore receptor	siderophore uptake transmembrane transporter, signaling receptor
A6739_RS06535	-	3.500019666	up	ABC transporter ATP-binding protein	ABC-type transporter, ATP binding, ATPase-coupled transmembrane transporter
A6739_RS07175	-	1.715529743	up	hypothetical protein	-
A6739_RS07555	*amdA*	1.461758079	up	amidase	amidase, indoleacetamide hydrolase
A6739_RS07560	-	1.364873266	up	acyl-CoA dehydrogenase	oxidoreductase
A6739_RS07580	-	1.127894768	up	aromatic-ring-hydroxylating dioxygenase subunit beta	dioxygenase
A6739_RS08375	*azr*	−1.071338077	down	FMN-dependent NADH-azoreductase	oxidoreductase
A6739_RS08405	-	−1.139044967	down	TIGR01244 family phosphatase	hydrolase, oxidoreductase
A6739_RS08410	-	−1.273424919	down	MBL fold metallo-hydrolase	beta-lactamase, protein dimerization, zinc ion binding
A6739_RS09455	-	−1.043166606	down	amino acid ABC transporter permease	transmembrane transporter
A6739_RS10785	-	1.769148258	up	hypothetical protein	-
A6739_RS11485	*plaP*	−1.747668925	down	APC family permease	transmembrane transporter
A6739_RS11675	-	1.162127898	up	hypothetical protein	-
A6739_RS12210	*nlpE*	1.518804309	down	copper resistance protein NlpE	-
A6739_RS14015	*pntB*	1.06376377	up	NAD(P) transhydrogenase subunit alpha	NAD(P)+ transhydrogenase, NAD(P) binding, protein dimerization
A6739_RS15535	-	1.132093844	up	hypothetical protein	-

## Data Availability

Not applicable.
